# Activation and Function of NLRP3 Inflammasome in Bone and Joint-Related Diseases

**DOI:** 10.3390/ijms23105365

**Published:** 2022-05-11

**Authors:** Tomohiko Murakami, Yuri Nakaminami, Yoshifumi Takahata, Kenji Hata, Riko Nishimura

**Affiliations:** Department of Molecular and Cellular Biochemistry, Osaka University Graduate School of Dentistry, Osaka 565-0871, Japan; n-yuri521@dent.osaka-u.ac.jp (Y.N.); takahata.yoshifumi.dent@osaka-u.ac.jp (Y.T.); hata@dent.osaka-u.ac.jp (K.H.); rikonisi@dent.osaka-u.ac.jp (R.N.)

**Keywords:** interleukin-1, inflammasome, NLRP3, caspase-1, rheumatoid arthritis, osteoarthritis, gout, osteoporosis, periodontal disease, aging, bone and joint diseases

## Abstract

Inflammation is a pivotal response to a variety of stimuli, and inflammatory molecules such as cytokines have central roles in the pathogenesis of various diseases, including bone and joint diseases. Proinflammatory cytokines are mainly produced by immune cells and mediate inflammatory and innate immune responses. Additionally, proinflammatory cytokines accelerate bone resorption and cartilage destruction, resulting in the destruction of bone and joint tissues. Thus, proinflammatory cytokines are involved in regulating the pathogenesis of bone and joint diseases. Interleukin (IL)-1 is a representative inflammatory cytokine that strongly promotes bone and cartilage destruction, and elucidating the regulation of IL-1 will advance our understanding of the onset and progression of bone and joint diseases. IL-1 has two isoforms, IL-1α and IL-1β. Both isoforms signal through the same IL-1 receptor type 1, but the activation mechanisms are completely different. In particular, IL-1β is tightly regulated by protein complexes termed inflammasomes. Recent research using innovative technologies has led to a series of discoveries about inflammasomes. This review highlights the current understanding of the activation and function of the NLRP3 (NOD-like receptor family, pyrin domain-containing 3) inflammasome in bone and joint diseases.

## 1. Introduction

Proinflammatory cytokines are important molecules that mediate inflammatory and innate immune responses and are involved in the development of inflammatory and chronic diseases [1,2,3], including bone and joint diseases such as rheumatoid arthritis (RA), osteoarthritis (OA), and periodontal disease [4,5,6]. Interleukin (IL)-1 is a typical proinflammatory cytokine and is critical for the inflammatory response, bone resorption, and cartilage destruction [7,8,9,10]. Additionally, IL-1 is involved in normal bone metabolism through osteoclastogenesis [11].

IL-1α and IL-1β are known isoforms of IL-1 [7,12]. Both IL-1α and IL-1β signal through IL-1 receptor type 1 (IL-1R1), which transmits the effects of IL-1 to cells. Thus, IL-1α and IL-1β are thought to have the same function and activity. IL-1 is produced predominantly by inflammatory and immune cells, and it has also been reported to be produced by other cell types such as keratinocytes, endothelial cells, epithelial cells, fibroblasts, and synoviocytes [7,12,13,14].

Although IL-1α and IL-1β have the same biological activity, their activation mechanisms are completely different [7,12]. IL-1 has pro and mature forms. Pro-IL-1α is cleaved by calpain to the mature form, and both pro-IL-1α and mature IL-1α are bioactive. However, pro-IL-1β is cleaved by caspase-1 (also termed IL-1β-converting enzyme; ICE) to the mature form, and mature IL-1β is biologically active, but pro-IL-1β is not. Activation of caspase-1 is tightly regulated by multiple protein complexes termed inflammasomes [15]. Thus, IL-1β signaling is controlled by inflammasomes.

IL-1α and IL-1β secretion is mediated by the same unique system using plasma membrane permeabilization [16,17]. Because both pro-IL-1α and pro-IL-1β are produced in the cytoplasm and not the endoplasmic reticulum (ER), IL-1α and IL-1β secretion requires necrotic cell death, which induces plasma membrane permeabilization. Indeed, caspase-1 activation by inflammasomes induces caspase-1-dependent necrotic cell death, termed pyroptosis. Therefore, inflammasomes contribute to IL-1α and IL-1β secretion through plasma membrane rupture triggered by pyroptosis [16,17]. Inflammasomes also induce maturation and secretion of IL-18 similarly to those of IL-1β [18]. Thus, the inflammasome regulates IL-1β and IL-18 maturation as well as pyroptosis, which contributes to IL-1α, IL-1β, and IL-18 secretion.

Recently, inflammasomes have been reported to be associated with the onset and progression of bone and joint diseases [19] as well as various other diseases [20,21]. In this review, we focus on the current understanding of the molecular mechanisms of inflammasomes and review the role of the NOD-like receptor family, pyrin domain-containing 3 (NLRP3) inflammasome in the pathogenesis of bone and joint diseases.

## 2. Inflammasomes

### 2.1. Representative Inflammasomes

**Figure 1:**
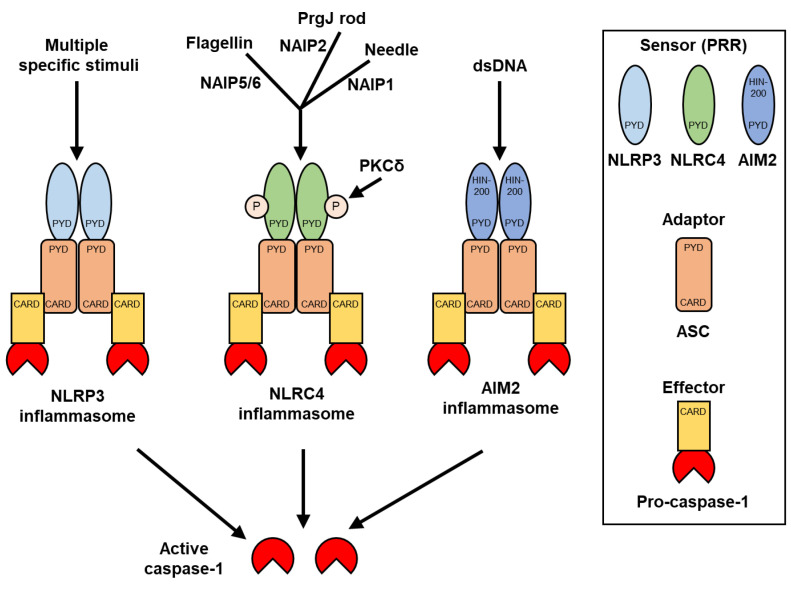
Components of NLRP3, NLRC4, and AIM2 inflammasomes. NLRP3, NLRC4, and AIM2 sense specific stimuli and induce assembly of inflammasomes with ASC and pro-caspase-1. Although NLRP3 reacts with multiple specific stimuli, NLRC4 reacts with cytosolic flagellin, PrgJ rod protein, and needle protein through NLR family, apoptosis inhibitory proteins (NAIPs), whereas AIM2 reacts with cytosolic dsDNA. The activated pattern recognition receptors (PRRs) interact with ASC via its pyrin domain (PYD). ASC interacts with caspase-1 via its caspase recruitment domain (CARD). Inflammasomes induce caspase-1 activation, which leads to both IL-1β and IL-18 maturation as well as cell death (pyroptosis). P indicates phosphorylation of NLRC4 by PKCδ.

Inflammasomes are molecular platforms for caspase-1 activation and are typically composed of three parts: a sensor protein, adapter protein, and pro-caspase-1 [15] (Figure 1). Inflammasome-composing pattern recognition receptors (PRRs) contain a pyrin domain (PYD) at the N-terminus. The PYD of PRRs interacts with the PYD of apoptosis-associated speck-like protein containing a caspase recruitment domain (CARD) (ASC). The CARD of ASC binds to the CARD of pro-caspase-1. Each PRR senses distinct pathogen-associated molecular patterns (PAMPs) or damage-associated molecular patterns (DAMPs). Activated PRR forms an inflammasome with ASC and pro-caspase-1, leading to caspase-1 activation.

NLRP3, NLR family CARD domain-containing protein 4 (NLRC4/IPAF), and absent in melanoma 2 (AIM2) are representative PRRs that form inflammasomes. The regulatory and activation mechanisms of NLRC4 and AIM2 are better understood compared with those of NLRP3.

NLRC4 recognizes cytosolic flagellin (fliC), a structural protein in bacterial flagella, through the NLR family, apoptosis inhibitory protein (NAIP) 5/6 [22] (Figure 1). NAIP5/6 directly binds to flagellin and interacts with NLRC4. Similarly, NLRC4 recognizes cytosolic PrgJ rod protein and needle protein, structurally related subunits of the type III secretion system (T3SS), through NAIP2 [23] and NAIP1 [24,25], respectively. In addition to the NAIP interaction with NLRC4, activation of the NLRC4 inflammasome is required for NLRC4 phosphorylation by PKCδ [26]. Therefore, NAIPs that recognize specific bacterial ligands and NLRC4 phosphorylation together promote NLRC4 inflammasome activation [27].

AIM2 consists of a C-terminal HIN-200 domain and N-terminal PYD [28,29] (Figure 1). The HIN-200 domain of AIM2 directly interacts with cytosolic double-stranded DNA (dsDNA) and forms the AIM2 inflammasome. AIM2 recognizes dsDNA of 80 bases or more in a sequence-independent manner [30]. Analysis of the AIM2 crystal structure revealed the activation mechanism of this DNA-sensing inflammasome. The HIN-200 domain and PYD of AIM2 form an intramolecular complex and maintain an autoinhibitory state [31]. When dsDNA binds to the HIN-200 domain, PYD is released from the intramolecular complex and interacts with the ASC. The sugar-phosphate backbone of dsDNA interacts with the positively charged HIN-200 domain via electrostatic attraction, allowing AIM2 to recognize DNA regardless of the sequence.

Unlike NLRC4 and AIM2, NLRP3 senses various stimuli, such as bacterial infection, extracellular ATP, crystals, lysosomal rupture, mitochondrial damage, and ER stress [32,33] (Figure 1). However, the activation of the NLRP3 inflammasome is tightly regulated similarly to that of NLRC4 and AIM2 inflammasomes. The NLRP3 inflammasome is associated with inflammatory and chronic diseases, including bone and joint diseases [19,20,21]. Human mutations in the *NLRP3* gene have been reported to cause an autoinflammatory disease known as cryopyrin-associated periodic syndrome (CAPS), which shows systematic and joint inflammation with hyperactivation of the NLRP3 inflammasome [34,35]. Therefore, understanding the regulatory mechanism of the NLRP3 inflammasome helps to elucidate the pathogenesis of these diseases.

### 2.2. Activation Mechanism of the NLRP3 Inflammasome

**Figure 2:**
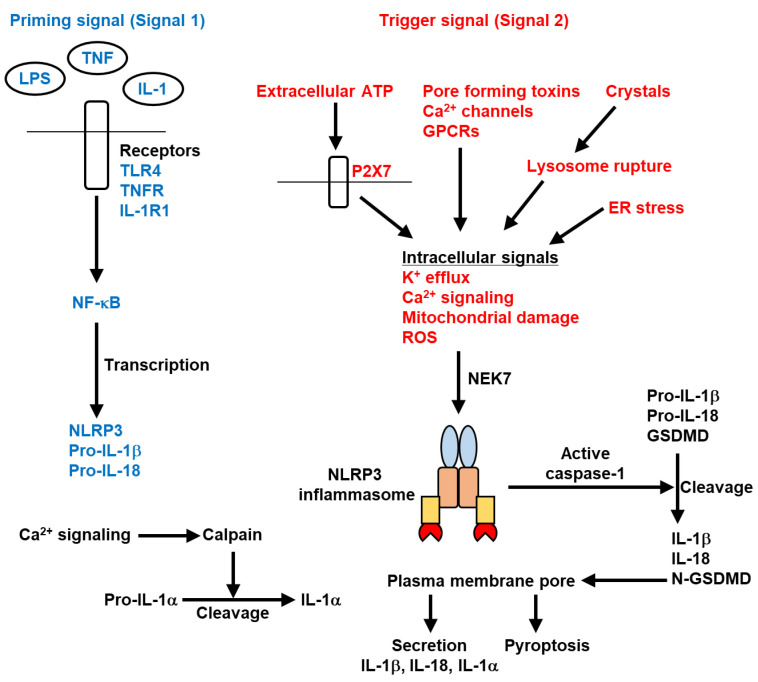
Mechanism of NLRP3 inflammasome activation. Pathogen-associated molecular patterns (PAMPs), such as lipopolysaccharide (LPS), or cytokines, such as TNFα and IL-1, induce a priming signal (Signal 1). The priming signal promotes expression of NLRP3, IL-1β, and IL-18 through NF-κB signaling. A trigger signal (Signal 2) is induced by specific PAMPs or damage-associated molecular patterns (DAMPs) such as bacterial infection, extracellular ATP, crystals, lysosomal rupture, mitochondrial damage, and endoplasmic reticulum (ER) stress, and leads to the assembly of NLRP3, ASC, and pro-caspase-1. Extracellular ATP stimulates P2X7 receptor. Phagocytosis of crystals induces lysosomal rupture. K^+^ efflux, Ca^2+^ signaling, mitochondrial damage, and reactive oxygen species (ROS) have been proposed to be the intracellular signals for NLRP3 inflammasome activation, while K^+^ efflux is thought to be the most important signal. These intracellular signals are provided by at least the P2X7 receptor, pore-forming toxins, Ca^2+^ channels, lysosomal rupture, ER stress, and G protein-coupled receptors (GPCRs). NEK7 is an essential positive regulator of the NLRP3 inflammasome and acts downstream of K^+^ efflux. The NLRP3 inflammasome activates caspase-1, inducing IL-1β and IL-18 maturation and GSDMD cleavage. Pro-IL-1α is cleaved by Ca^2+^-dependent protease calpain and becomes mature IL-1α. N-terminal fragments of GSDMD (N-GSDMD) insert into the plasma membrane and form pores, leading to pyroptosis and secretion of IL-1β, IL-18, and IL-1α. Blue and red words indicate Signal 1 and Signal 2, respectively.

Activation of the NLRP3 inflammasome requires a two-step process termed Signal 1 and Signal 2 [32,33] (Figure 2). Signal 1 is priming at transcriptional and post-translational levels, which is triggered by specific PAMPs, such as lipopolysaccharide (LPS), or cytokines, such as TNFα and IL-1. The priming signal leads to transcriptional activation of NLRP3 inflammasome-related genes, such as NLRP3, pro-IL-1β, and pro-IL-18, through activation of the nuclear factor-κB (NF-κB) signaling pathway. Signal 2 is the trigger step for activation and is induced by specific PAMPs or DAMPs, such as bacterial infection, extracellular ATP, crystals, lysosomal rupture, mitochondrial damage, and ER stress. The trigger signal induces the assembly of NLRP3, ASC, and pro-caspase-1, leading to the activation of the NLRP3 inflammasome.

NLRP3 contains PYD, NACHT, and leucine-rich repeat (LRR) domains [15]. Because it is unlikely that NLRP3 or these domains directly sense all stimuli that activate the NLRP3 inflammasome, NLRP3 is thought to sense common intracellular signals induced by these stimuli. K^+^ efflux, Ca^2+^ signaling, mitochondrial damage, and reactive oxygen species (ROS) have been proposed as intracellular signals for NLRP3 inflammasome activation [36,37,38,39,40] (Figure 2). Indeed, activation of the NLRP3 inflammasome is inhibited by the blockade of intracellular signals such as K^+^ efflux, Ca^2+^ signals, and ROS. These intracellular signals are mediated by at least the P2X7 receptor, pore-forming toxins, lysosomal rupture, Ca^2+^ channels, mitochondrial damage, ER stress, and G protein-coupled receptors (GPCRs) [32,33,41,42]. Among these signals, K^+^ efflux is the most important for NLRP3 inflammasome activation [43], although it has been reported that some conditions for NLRP3 activation are independent of K^+^ efflux [44,45].

NIMA-related kinase 7 (NEK7) is a critical regulator for NLRP3 activation downstream of K^+^ efflux [46,47,48] (Figure 2). NEK7 directly binds to NLRP3 to mediate NLRP3 inflammasome assembly and activation. Cryoelectron microscopy has revealed that NEK7 bridges adjacent NLRP3 subunits in two interactions and mediates the activation of the NLRP3 inflammasome [49]. Additionally, NEK7 is required for the hyperactivation of the CAPS-associated NLRP3 mutation to activate caspase-1 [46]. However, the exact mechanisms by which NEK7 senses K^+^ efflux and binds to NLRP3 remain unclear. Therefore, understanding the molecular mechanisms that link NEK7 to K^+^ efflux would facilitate elucidating the precise mechanism of NLRP3 inflammasome activation.

In addition to these positive regulators of the NLRP3 inflammasome, Gasdermin D (GSDMD) has been reported to be an essential regulator for cell death induced by caspase-1 [50,51] (Figure 2). Activation of caspase-1 by inflammasomes induces GSDMD cleavage. The N-terminal fragments of cleaved GSDMD insert into the plasma membrane and form large permeability pores that cause cell death. IL-1β secretion is suppressed in GSDMD-deficient macrophages even if active caspase-1 promotes IL-1β maturation. Therefore, GSDMD-dependent cell death is required for IL-1β secretion.

Regarding cell death induced by the NLRP3 inflammasome, DEAD-Box Helicase 3 X-Linked (DDX3X) [52] has been reported to be a unique modulator of cell survival or death. DDX3X is a component of stress granules, cytoplasmic compartments that assemble in response to various stresses and contribute to survival [53]. DDX3X interacts with NLRP3 to promote NLRP3 inflammasome activation. Conversely, the formation of stress granules incorporates DDX3X, resulting in the inhibition of NLRP3 inflammasome activation [52]. Thus, DDX3X acts as a check point for cell survival or death by modulating NLRP3 inflammasome activation.

### 2.3. Negative Regulation of the NLRP3 Inflammasome

Because negative regulation of the NLRP3 inflammasome is a potential therapeutic target for NLRP3-associated diseases, negative regulators of the NLRP3 inflammasome have been investigated [54]. In particular, intracellular degradation systems, such as autophagy and ubiquitination, are associated with the negative regulation of the NLRP3 inflammasome.

Autophagy is an intracellular degradation system that delivers cytoplasmic material and organelles to the lysosome [55]. Loss of autophagy by Atg16L1 deficiency enhances NLRP3 inflammasome activation [56]. Autophagy removes damaged mitochondria via mitophagy [40,57] and degrades ubiquitinated NLRP3 inflammasome complexes [58], resulting in the negative regulation of NLRP3 inflammasome activation. NLRP3 tyrosine phosphorylation (Tyr861) has also been reported to be a negative regulator of the NLRP3 inflammasome in an autophagy-dependent manner [59,60]. NLRP3 tyrosine phosphorylation is controlled by PTPN22 (protein tyrosine phosphatase nonreceptor 22), and its deficiency in mice increases NLRP3 tyrosine phosphorylation and reduces the mature IL-1β level.

The ubiquitin–proteasome system is also associated with the negative regulation of the NLRP3 inflammasome [54]. Ubiquitinated NLRP3 is degraded by the proteasome, whereas BRISC (Brcc36 isopeptidase complex), a deubiquitinating enzyme complex, promotes NLRP3 inflammasome activation by deubiquitination of NLRP3 [61,62]. Several E3 ubiquitin ligases, such as MARCH7 [63], TRIM31 [64], FBXL2 [65], and ARIH2 [66], have been reported to promote NLRP3 ubiquitination. Dopamine D1 receptor (DRD1) promotes NLRP3 ubiquitination by MARCH7 [63]. Additionally, phosphorylation of NLRP3 (S295) has been reported to be a negative regulator of the NLRP3 inflammasome through ubiquitination of NLRP3 [67]. The transmembrane G protein-coupled receptor 5 (TGR5) bile acid receptor activates protein kinase A (PKA). Active PKA induces the ubiquitination of NLRP3 via NLRP3 phosphorylation.

Because DRD1 and TGR5 are GPCRs, G protein signals are also involved in the negative regulation of the NLRP3 inflammasome. GPSM3 (G protein signaling modulator-3) suppresses NLRP3 inflammasome activation by interacting with the C-terminal LRR domain of NLRP3 [68]. RalB (Ras-like small G protein) inhibits NLRP3 by promoting the formation of autophagosomes [58]. G protein subunit β1 (GNB1/Gβ1), which is involved in the development of the neural tube and endochondral ossification [69,70], negatively regulates the NLRP3 inflammasome [71]. GNB1 suppresses NLRP3-induced ASC oligomerization, which is essential for caspase-1 activation. However, several GPCRs are positive regulators of the NLRP3 inflammasome, including calcium-sensing receptor (CASR) and GPRC6A (G protein-coupled receptor family C group 6 member A) [38,72]. Therefore, G protein signaling may contribute to balancing the negative and positive regulation of the NLRP3 inflammasome.

### 2.4. Non-Canonical NLRP3 Inflammasome

**Figure 3:**
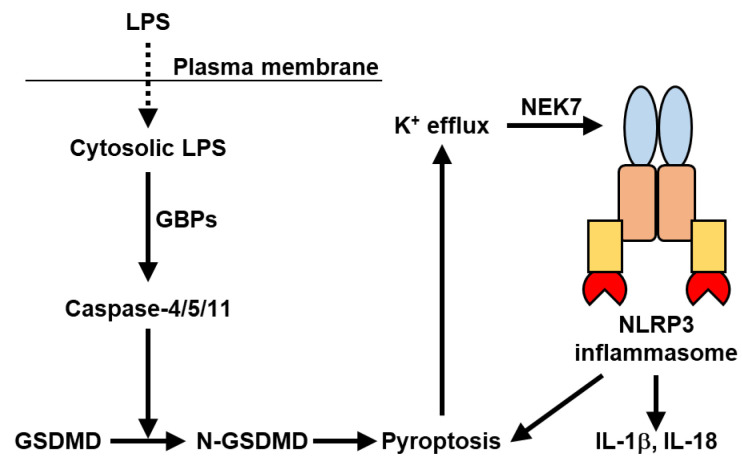
Mechanism of non-canonical NLRP3 inflammasome activation. Caspase-4/5/11 sense cytosolic lipopolysaccharide (LPS) from Gram-negative bacteria. Guanylate-binding proteins (GBPs) bind to cytosolic LPS and are implicated in the activation of the caspases. Active caspase-4/5/11 directly cleave GSDMD independently of caspase-1. Cleaved N-terminal fragments of GSDMD (N-GSDMD) induce pyroptosis, which triggers K^+^ efflux and activation of the non-canonical NLRP3 inflammasome. Activated caspase-1 induces IL-1β and IL-18 maturation and promotes pyroptosis. NEK7 is required for the non-canonical NLRP3 inflammasome downstream of K^+^ efflux.

While studying the response of inflammasomes to cholera toxin B (CTB), the non-canonical NLRP3 inflammasome pathway was discovered [73,74]. The non-canonical NLRP3 inflammasome pathway is regulated by caspase-11 (Figure 3). Activation of caspase-11 by CTB in LPS-primed macrophages is triggered by intracellular delivery of LPS by CTB. Indeed, transfection or electroporation of LPS, which delivers LPS into the cytosol, induces the activation of the non-canonical NLRP3 inflammasome pathway [75,76]. Caspase-11 is critical for caspase-1 activation and IL-1β production in macrophages infected with *Escherichia coli*, *Citrobacter rodentium*, or *Vibrio cholerae*.

In addition to caspase-11, human caspase-4 (caspase-11 in mice) and caspase-5 activate the non-canonical NLRP3 inflammasome pathway [18] (Figure 3). These caspases sense cytosolic LPS from Gram-negative bacteria. Activated caspase-4, caspase-5, and caspase-11 directly promote pyroptosis through the cleavage of GSDMD, but they do not cleave pro-IL-1β or pro-IL-18 [73,74,77]. Pyroptosis induces K^+^ efflux, which triggers the activation of the non-canonical NLRP3 inflammasome for IL-1β and IL-18 maturation. NEK7 is required for the non-canonical NLRP3 inflammasome downstream of K^+^ efflux. Recent studies have shown that several guanylate-binding proteins (GBPs) bind to cytosolic LPS and are implicated in caspase-4 activation [78,79,80,81].

In addition to cytosolic LPS, oxidized phospholipid 1-palmitoyl-2-arachidonoyl-sn-glycero-3-phosphorylcholine (oxPAPC), which modulates inflammatory responses, has also been reported to bind to caspase-11 and activate the non-canonical pathway [82]. Cytoplasmic LPS induces IL-1β release and pyroptosis via caspase-11, whereas oxPAPC induces only IL-1β release without cell death in dendritic cells. These differences may be due to the different binding sites in caspase-11 for LPS and oxPAPC, which promote different enzymatic activities of caspase-11. LPS binds to the CARD of caspase-11 and induces its enzymatic activity, whereas oxPAPC binds to the catalytic domain and inhibits the catalytic domain. However, oxPAPC inhibits the non-canonical inflammasome in macrophages [83]. Therefore, the precise role of oxPAPC in the non-canonical inflammasome remains controversial.

## 3. NLRP3 Inflammasome in Bone and Joint Diseases

### 3.1. Rheumatoid Arthritis (RA)

RA is a chronic autoinflammatory arthritis that leads to joint malformations and dysfunction [84]. Proinflammatory cytokines including IL-1 are crucial for RA pathogenesis [4,85]. IL-1β is expressed in synovial tissue and activates osteoclasts and chondrocytes, leading to bone and cartilage destruction [86,87]. IL-1β also promotes the differentiation of Th17 (T helper type 17) cells, which contributes to RA pathogenesis [88]. Additionally, mice deficient for an endogenous IL-1 inhibitor, IL-1 receptor antagonist (IL-1Ra), spontaneously develop arthritis. [89].

The NLRP3 inflammasome is involved in the pathogenesis and development of RA [90], and polymorphisms in the *NLRP3* gene correspond to RA susceptibility [91,92,93,94]. Clinical studies have found elevated NLRP3 protein expression in peripheral blood cells, monocytes, macrophages, and dendritic cells of RA patients [4,95,96], suggesting that NLRP3 activation is associated with the systemic and local inflammation of RA.

Various RA model mice have been analyzed in the field of inflammasomes. The NLRP3 inflammasome is activated in synovial tissues of collagen-induced arthritis (CIA) mice [90]. Expression of NLRP3, caspase-1, and IL-1 is increased in the synovial tissues of CIA mice, and IL-1α, IL-1β, and IL-1αβ-deficient mice are less sensitive to CIA-induced arthritis [97]. Additionally, IL-18-deficient mice are less sensitive to CIA-induced arthritis [98]. Altered expression of the NF-κB suppressor gene *A20/TNFAIP3* has been found in monocytes from RA patients [99], and myeloid-cell-specific deletion of *A20* in mice triggers spontaneous arthritis [100]. NLRP3 deficiency in this mouse model suppresses the development of inflammation and cartilage destruction [101]. The CASR-mediated NLRP3 inflammasome is also involved in RA [102]. Anti-inflammatory cytokine IL-10-deficient mice exhibit severe antigen-induced arthritis with the upregulated expression of IL-1β and NLRP3 in the synovium [103]. However, there are controversial reports about NLRP3 and RA. ASC deficiency suppresses arthritis in RA model mice through reduced T cell priming, which is independent of NLRP3 or caspase-1 [104,105]. Therefore, the precise pathogenesis of RA involving the NLRP3 inflammasome requires further study.

### 3.2. Osteoarthritis (OA)

OA is a progressive disease of joints and the most common type of arthritis, which is characterized by the degradation of articular cartilage [106,107]. The exact mechanism of cartilage degradation in OA remains unclear, but is thought to be caused by mechanical stress and inflammation in joints. IL-1β has been implicated in the pathogenesis of OA by mediating synovial inflammation and cartilage degeneration [5,108]. IL-1β levels were more elevated in synovial fluid cells of OA patients than in those of controls [109], although IL-1β levels were lower in OA patients than in RA patients [110]. IL-1β promotes the production of proteases, such as metalloproteinases (MMPs) and aggrecanases, which degrade cartilage. The aggrecanases ADAMTS4 and ADAMTS5 are major enzymes in articular cartilage degradation of OA [111,112]. IL-1-induced ADAMTS4 and ADAMTS5 are controlled by SOX4 and SOX11, members of the SoxC family of transcription factors [113,114,115]. SOX4 and SOX11 expression is associated with the destruction of cartilage in OA patients, suggesting the importance of IL-1 signaling in OA progression.

NLRP3 protein expression was increased in patients with knee OA compared with controls in samples from the synovial membrane [116]. Furthermore, NLRP3 inflammasome activation has been detected in the synovium of OA patients and OA model animals [117]. These reports indicate that ROS in OA synovial tissues are involved in the activation of the NLRP3 inflammasome. In addition to ROS, ectopic deposition of hydroxyapatite (HA) crystals in joints is associated with OA pathogenesis [118]. HA crystals induce the secretion of IL-1β and IL-18 in macrophages in an NLRP3 inflammasome-dependent manner. Mice lacking NLRP3 or caspase-1 have reduced joint lesions with spontaneous HA deposition in a progressive ankylosis-deficient model of arthritis. Moreover, calcium crystals from synovial fluids of OA patients exhibit NLRP3 inflammasome-stimulatory activity in vitro [118]. These reports indicate the importance of the NLRP3 inflammasome in OA pathogenesis, although a report has suggested that OA pathogenesis is independent of the NLRP3 inflammasome [119].

Recent studies have reported that ER stress is associated with OA development [120,121]. Because ER stress induces NLRP3 inflammasome activation, ER stress in bone and cartilage tissues may be involved in OA pathogenesis through NLRP3 inflammasome activation. Indeed, osteoblasts and chondrocytes produce large amounts of bone and cartilage matrix, resulting in physiological ER stress [122,123,124]. Further studies are required to evaluate the relationship between ER stress and the NLRP3 inflammasome in OA pathogenesis.

### 3.3. Crystal-Induced Gout

Gout is an inflammatory arthritis that causes pain and swelling in joints due to monosodium urate (MSU) crystals [125]. Excess purines are normally degraded to uric acid, which is excreted in the urine. However, if excess purines are produced or if uric acid is not excreted sufficiently, the amount of uric acid in the body increases. The uric acid crystallizes as MSU and accumulates in joints, resulting in gout.

MSU crystals, which enhance IL1β release in synergy with LPS [126], activate the NLRP3 inflammasome, resulting in the production of active IL-1β and IL-18 [127]. IL-1R signaling is essential for the gouty inflammation induced by MSU [128]. Additionally, an association between *NLRP3* polymorphisms and susceptibility to gouty arthritis has been reported [129]. Pseudogout is an acute form of arthritis caused by calcium pyrophosphate dihydrate (CPPD) crystals without an increase in uric acid [130]. CPPD crystals are also involved in the activation of the NLRP3 inflammasome [127]. The major role of IL-1β in gout and pseudogout has been confirmed by the efficacy of blocking agents against IL-1 signaling. Anakinra, IL-1Ra recombinant, and canakinumab, an anti-IL-1β monoclonal antibody, have been found to reduce acute arthritis [131,132,133].

### 3.4. Osteoporosis

Osteoporosis is characterized by a decrease in bone mass, which increases bone fragility and fracture risk [134]. It is caused by an imbalance in bone metabolism toward bone resorption. Osteoporosis is particularly common in postmenopausal women and closely related to decreases in female hormones and aging [135,136,137]. Declining levels of estrogen and aging promote low levels of inflammation in the body, and the generated inflammatory cytokines are associated with osteoporosis by affecting osteoblast and osteoclast activities [138,139].

The levels of inflammatory cytokines are elevated in the absence of estrogen [140,141], and IL-1β plays a major role in bone loss associated with estrogen deficiency [142,143]. Blocking IL-1 in postmenopausal women reduces the expression of bone resorption markers [144]. NLRP3 deficiency prevents bone loss due to ovariectomy (OVX) in mice [145]. An in vivo study using NLRP3-deficient mice revealed that NLRP3 is involved in bone edification through the regulation of hypertrophic chondrocyte maturation and osteoblast activity [146]. Constitutively activated NLRP3 inflammasomes lead to abnormal skeletal development in mice [147]. Humanized mice expressing NLRP3 disease-associated mutations develop normally but show acute sensitivity to endotoxins and develop progressive and debilitating arthritis characterized by granulocytic infiltration, elevated cytokines, bone erosion, and osteoporosis [148]. Myeloid cell type–specific expression of an NLRP3 mutant revealed the role of the NLRP3 mutation in osteolysis [149]. The NLRP3 inflammasome in myeloid cells indirectly regulates osteoclast activity through systemic inflammation, whereas the NLRP3 inflammasome in osteoclasts directly regulates osteoclast activity through PARP1 proteolysis, a negative regulator of osteoclast formation. IL-18-binding protein (IL-18BP) is an antagonist of IL-18, and high serum IL-18BP is associated with a low risk of osteoporosis in postmenopausal women [150]. Modulation of the NLRP3 inflammasome pathway by IL-18BP promotes osteoblast differentiation and restores bone volume in OVX mice [150]. ASC contributes to osteoblast differentiation and osteogenesis [151]. Thus, the NLRP3 inflammasome is involved in both bone resorption and bone formation. Taken together, these studies indicate that the NLRP3 inflammasome plays a dual role in bone metabolism, and its abnormal activation affects the development of osteoporosis. Regulating the NLRP3 inflammasome would be a promising therapeutic strategy to prevent and control osteoporosis [152].

### 3.5. Periodontal Disease

Prolonged bacterial infection of periodontal tissues induces periodontitis, a condition that affects gums and alveolar bone [153]. The inflammatory response of periodontitis results in the infiltration of immune cells, such as macrophages and T cells, and the production of proinflammatory cytokines. These cytokines promote the differentiation and activation of osteoclasts that are responsible for bone resorption and cause alveolar bone destruction [6,9,153]. Thus, proinflammatory cytokines play a major role in the development and progression of periodontal disease.

Among the proinflammatory cytokines, IL-1 strongly induces bone destruction by promoting osteoclast differentiation and activity [154,155,156]. Moreover, clinical studies have shown that IL-1 levels in the gingival crevicular fluid of patients with periodontitis are significantly increased compared with those in controls, and that IL-1 levels are closely related to periodontal disease severity [157]. Additionally, transgenic mice overexpressing IL-1α spontaneously develop periodontal disease [158], and periodontitis mouse models deficient for IL-1Ra show severe periodontal disease progression [159]. These findings strongly suggest that IL-1 is a critical factor in the pathogenesis of periodontal disease by promoting alveolar bone destruction with the induction of inflammatory responses.

*Porphyromonas gingivalis, Treponema denticola,* and *Aggregatibacter actinomycetemcomitans* have been implicated in the onset and development of periodontal disease [160]. Infection with these bacteria activates the NLRP3 inflammasome [161,162,163], indicating that the NLRP3 inflammasome is associated with the pathogenesis of periodontal disease [164]. Indeed, a clinical study reported significantly higher expression of NLRP3 in gingival tissues of patients with periodontal disease compared with controls [165]. Moreover, a positive correlation was observed between the expression levels of NLRP3 and IL-1β in gingival tissues. Another clinical study showed that saliva samples from a periodontitis group had significantly higher levels of NLRP3 and IL-1β than controls [166]. Additionally, IL-18 secretion has been associated with periodontal disease progression [167]. Inhibition of the NLRP3 inflammasome by shRNA targeting NLRP3 increases the expression of osteogenic markers and promotes healing of alveolar bone defects in diabetic rats [168]. Activation of the NLRP3 inflammasome has also been reported in human dental pulp tissue and fibroblasts [169]. Taken together, these studies support that the NLRP3 inflammasome is involved in the pathogenesis of periodontal disease. Furthermore, periodontal disease is considered to be a risk factor for other NLRP3 inflammasome-related diseases, such as RA, diabetes, and Alzheimer’s disease [170,171,172]. Therefore, chronic inflammation and/or pathogens in periodontal disease might play a role in accelerating the development of these diseases through IL-1 and NLRP3 inflammasome activation.

### 3.6. Aging

Aging may be associated with a low level of chronic inflammation, indicated by elevated levels of proinflammatory cytokines [173]. It is known that aging induces bone loss [174]. NLRP3 inflammasome has been reported to be involved in bone loss during aging [175]. The NLRP3 inflammasome controls systemic low-grade age-related inflammation, and aged NLRP3-deficient mice have increased cortical and trabecular bone volumes compared with aged WT mice. Additionally, NLRP3-deficient mice do not show a decrease in the bone marrow volume, indicating that the increased bone volumes in NLRP3-deficient mice are not caused by osteoporosis development due to osteoclast dysfunction. However, aged IL-1R-deficient animals are not protected from bone loss caused by aging [175]. Despite the important role of IL-1 in bone homeostasis, bone loss during healthy aging is dependent on NLRP3, but not on IL-1, signaling. Furthermore, aged caspase-11-deficient mice are not protected from bone loss caused by aging, indicating that bone loss during aging does not involve non-canonical inflammasomes [175].

Because an increase in cortical bone mass has been demonstrated to be a predictor of an enhanced lifespan in mice [176], attenuation of age-related degenerative changes mediated by inflammasomes might promote longevity. Indeed, inflammasome modules have correlated with health and longevity in some cohorts [177]. Another basic study showed that inhibition of the NLRP3 inflammasome reduces age-related alveolar bone loss through the suppression of osteoclast differentiation and caspase-1 activation [178]. This phenomenon was not observed in young mice. Further study is required to assess whether inhibition of the NLRP3 inflammasome can prevent age-related bone loss and promote longevity.

## 4. Conclusions

The NLRP3 inflammasome is involved in the pathogenesis of bone and joint diseases by affecting inflammation, bone resorption, and bone formation. Because the NLRP3 inflammasome and IL-1 signaling pathways are critical factors in the onset and progression of various inflammatory and chronic diseases, blockade of these signaling pathways is an effective therapeutic strategy for the treatment or prevention of NLRP3 inflammasome-related diseases. Indeed, several inhibitors of the IL-1 signaling pathway have already been developed and approved for the treatment of several diseases [179]. For example, anakinra is a recombinant IL-1Ra that has been approved for RA treatment [180], and canakinumab is a human anti-IL-1β monoclonal antibody used for CAPS treatment [181].

In addition to IL-1 signaling inhibitors, inflammasome inhibitors have been developed. VX-765 is a caspase-1 inhibitor [182]. VX-765 reduces disease severity and the expression of IL-1β and IL-18 in animal models of RA, skin inflammation, and epilepsy [183,184]. VX-765 was tested in a phase II clinical trial for drug-resistant partial epilepsy, but the results showed no statistically significant difference between VX-765 and placebo groups [182]. MCC950 is an NLRP3 inflammasome inhibitor that inhibits the oligomerization of ASCs required for NLRP3 inflammasome activation [185]. The efficacy of MCC950 was tested in a phase II clinical trial for RA, but the trial was terminated because of liver toxicity [186]. Recently, other NLRP3 inflammasome inhibitors have also been developed [187]. CY-09 directly binds to the ATP-binding motif of the NLRP3 NACHT domain and inhibits NLRP3 ATPase activity, which is essential for the oligomerization of NLRP3 [188]. OLT1177 reduces the ATPase activity of NLRP3, resulting in the suppression of NLRP3 inflammasome activation [189]. Tranilast blocks the oligomerization of NLRP3 [190]. Oridonin blocks the interaction between NLRP3 and NEK7, thereby inhibiting NLRP3 inflammasome activation [191]. Further studies and clinical trials might clarify the efficacy of these inhibitors and lead to the development of NLRP3 inflammasome inhibitors for the treatment or prevention of bone and joint diseases.

## Data Availability

Not applicable.
